# Variants in mitochondrial disease genes are common causes of inherited peripheral neuropathies

**DOI:** 10.1007/s00415-024-12319-y

**Published:** 2024-03-28

**Authors:** Tomas Ferreira, Kiran Polavarapu, Catarina Olimpio, Ida Paramonov, Hanns Lochmüller, Rita Horvath

**Affiliations:** 1 Department of Clinical Neurosciences, John Van Geest Centre for Brain Repair, School of Clinical Medicine, University of Cambridge, Robinson Way, Cambridge, CB2 0PY UK; 2 Children’s Hospital of Eastern Ontario Research Institute, Ottawa, ON Canada; 3 East Anglian Medical Genetics Service, Cambridge University Hospitals NHS Foundation Trust, Cambridge, UK; 4 Centro Nacional de Análisis Genómico, Barcelona, Spain; 5 Division of Neurology, Department of Medicine, The Ottawa Hospital, Ottawa, Canada; 6 Brain and Mind Research Institute, University of Ottawa, Ottawa, Canada; 7 Department of Neuropediatrics and Muscle Disorders, Faculty of Medicine, Medical Center - University of Freiburg, Freiburg, Germany

**Keywords:** Peripheral neuropathies, CMT, Mitochondrial disease, Genome-phenome analysis platform (GPAP), Rare variants, Genetic heterogeneity

## Abstract

**Background:**

Peripheral neuropathies in mitochondrial disease are caused by mutations in nuclear genes encoding mitochondrial proteins, or in the mitochondrial genome. Whole exome or genome sequencing enable parallel testing of nuclear and mtDNA genes, and it has significantly advanced the genetic diagnosis of inherited diseases. Despite this, approximately 40% of all Charcot-Marie-Tooth (CMT) cases remain undiagnosed.

**Methods:**

The genome-phenome analysis platform (GPAP) in RD-Connect was utilised to create a cohort of 2087 patients with at least one Human Phenotype Ontology (HPO) term suggestive of a peripheral neuropathy, from a total of 10,935 patients. These patients’ genetic data were then analysed and searched for variants in known mitochondrial disease genes.

**Results:**

A total of 1,379 rare variants were identified, 44 of which were included in this study as either reported pathogenic or likely causative in 42 patients from 36 families. The most common genes found to be likely causative for an autosomal dominant neuropathy were *GDAP1* and *GARS1*. We also detected heterozygous likely pathogenic variants in *DNA2, MFN2*, *DNM2, PDHA1, SDHA,* and *UCHL1*. Biallelic variants in *SACS, SPG7*, *GDAP1, C12orf65, UCHL1, NDUFS6, ETFDH* and *DARS2* and variants in the mitochondrial DNA (mtDNA)-encoded *MT-ATP6* and *MT-TK* were also causative for mitochondrial CMT. Only 50% of these variants were already reported as solved in GPAP.

**Conclusion:**

Variants in mitochondrial disease genes are frequent in patients with inherited peripheral neuropathies. Due to the clinical overlap between mitochondrial disease and CMT, agnostic exome or genome sequencing have better diagnostic yields than targeted gene panels.

**Supplementary Information:**

The online version contains supplementary material available at 10.1007/s00415-024-12319-y.

## Introduction

Inherited peripheral neuropathies or Charcot-Marie-Tooth disease (CMT) is a group of closely related disorders affecting the motor and sensory neurones of the peripheral nervous system. With an estimated prevalence of 40 individuals per 100,000, CMT is among the most common hereditary neuromuscular disorders. CMT is categorised into demyelinating and axonal subtypes, based on the primary mechanism of degeneration [1, 2].

CMT typically presents in the first or second decade of life, but the age of onset can vary widely, ranging from early infancy to late adulthood. Typical symptoms include symmetrical distal weakness and atrophy, sensory loss, foot deformities (pes cavus or planus, hammer toes, and clawing fingers), and reduced or absent tendon reflexes. Sensorineural hearing loss and phrenic nerve mediated respiratory insufficiency have also been reported. For the vast majority of patients, these symptoms are painless and, although progressively debilitating, not life threatening [3].

CMT exhibits significant genetic heterogeneity, with over 100 causative genes identified to date, and can be inherited through several different modes of inheritance, including autosomal dominant, autosomal recessive, X-linked, and mitochondrial. These genes encompass a wide range of functions, including those involved in peripheral nerve structure, myelin production, axonal transport, and mitochondrial dynamics [4, 5]. This genetic diversity contributes to the significant phenotypic variability observed in CMT, with variations in age of onset, disease progression, severity of symptoms, and specific clinical features. Understanding the underlying genetic subtypes and their associated phenotypic variations is crucial for accurate diagnosis, prognosis, and personalised management strategies. Clinical diagnosis is complex and often elusive, focusing on clinical presentation, neurophysiological tests, genetic and molecular tests, and previously on nerve biopsy. The introduction of next-generation sequencing (NGS) technology has significantly advanced the genetic diagnosis of inherited rare diseases in recent years [6]. Despite this, in approximately 40% of all CMT cases a genetic diagnosis is not identified [7].

The normal functioning of neurons and their long axons is dependent on healthy mitochondria, which play critical roles in axonal transport, energy production, calcium buffering, and other essential cellular functions. The distribution of mitochondria along peripheral axons is regulated by a continuous process associated with mitochondrial fusion and fission, which is collectively known as mitochondrial dynamics [8]. Disruptions to mitochondria have been increasingly recognised as a cause of peripheral nerve dysfunction. In keeping with this, a third of patients with mitochondrial disorders develop peripheral neuropathy [9–11]. Peripheral neuropathies in mitochondrial disease are typically the result of mutations in genes that are either nuclear encoded, and whose products are subsequently transported to the mitochondria, or in the mitochondrial genome itself.

Reanalysis of existing sequencing data provides an opportunity to enhance the diagnostic yield by leveraging improved bioinformatics pipelines and updated literature [12–29]. Previous studies have demonstrated that data reanalysis or re-evaluation can significantly increase the diagnostic yield, depending on the timing of the initial analysis and the interval before reanalysis. The American College of Medical Genetics and Genomics (ACMG) has outlined guidelines for variant-level re-evaluation and case-level reanalysis of genomic test results [30]. Keeping phenotypic descriptions comprehensive and up to date is recommended, as it can improve the specificity of the phenotype and contribute to increased diagnostic success for unsolved cases.

The RD-Connect Genome-Phenome Analysis Platform (GPAP) is an online platform (accessible at https://platform.rd-connect.eu/) designed to facilitate the analysis of genome-phenome data for rare disease (RD) diagnosis and gene discovery [31]. The platform includes heterogeneous datasets, contributed by various clinical researchers, generated in different clinical centres and genomic facilities. To ensure consistency and standardisation across all patients and their relatives, the GPAP platform incorporates a mechanism for submitting pseudonymised phenotypic data using recognised ontologies and standards including the Human Phenotype Ontology (HPO) [32], the Orphanet Rare Disease Ontology (ORDO) [33], and the Online Mendelian Inheritance in Man database (OMIM) [34]. By harmonising the information using these ontologies, the GPAP enhances interoperability and facilitates comprehensive analysis by standardised pipelines. WES/WGS raw data (fastq files) are also processed through a standardised pipeline making sequencing results between different facilities, kits, etc., better comparable.

In our study we aimed to identify a genetic diagnosis on a cohort of patients with possible inherited peripheral neuropathy, with particular focus on mitochondrial genes through the RD-Connect Platform.

## Methods

### Selection criteria

**Fig. 1:**
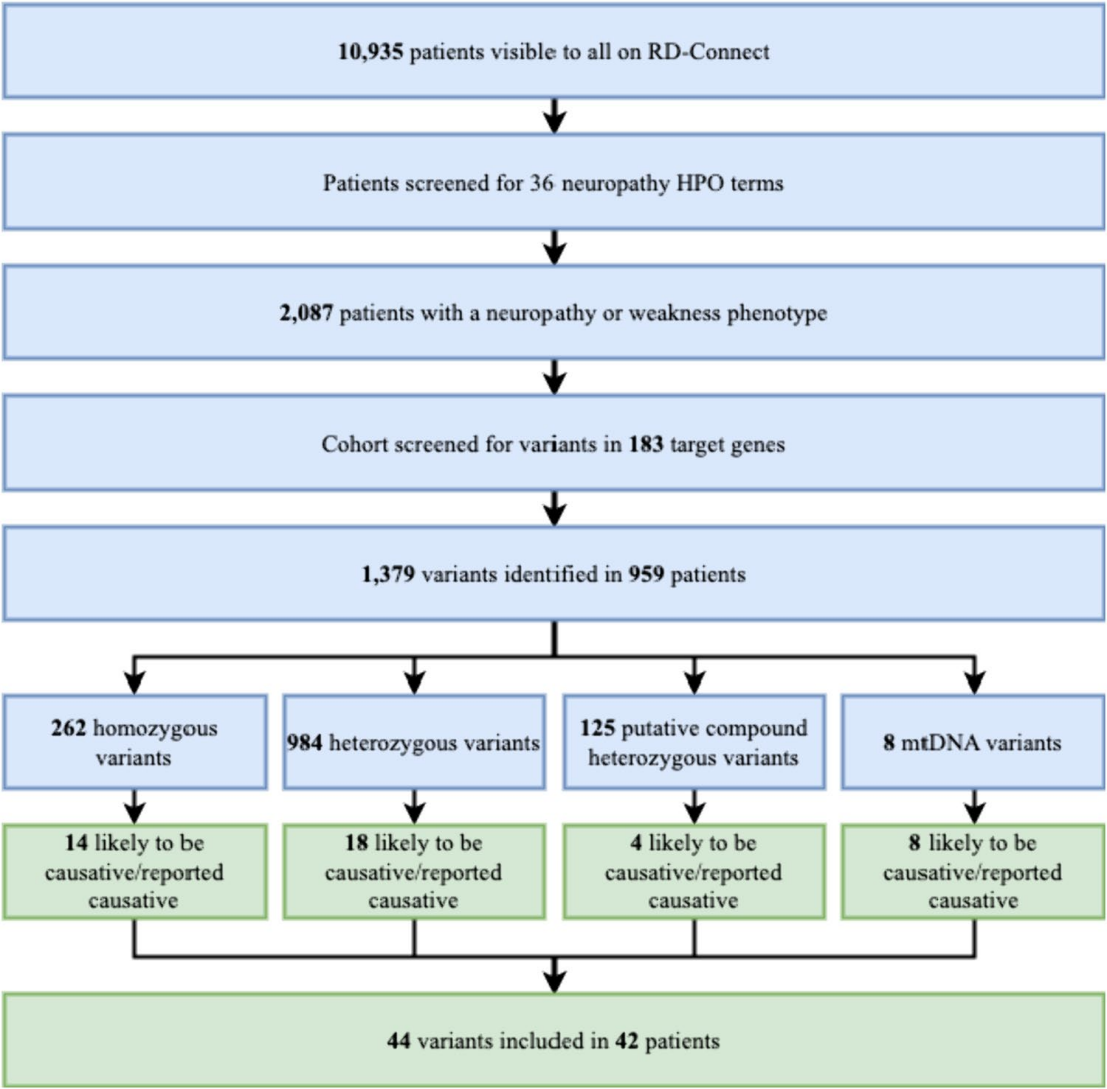
Study algorithm – highlighting the steps taken in the study process

A total of 10,935 individuals (patients and relatives) were visible to all registered and authorised users on the RD-Connect GPAP platform in January 2023. Clinicians uploading patient genomic records on the platform are asked to phenotypically characterise the individuals through HPO terms. We compiled a list of HPO terms commonly associated with inherited neuropathies to create a virtual peripheral neuropathy cohort within GPAP (see Supplemental Materials for the complete list). A search for individuals within the platform with any of these HPO terms resulted in 2087 patients forming our inherited peripheral neuropathy cohort, but not all of these patients have been diagnosed with peripheral neuropathy as a symptom like distal weakness or areflexia could have a different cause (e.g. distal myopathy) **(**Fig. 1**).**

### Target gene selection

To identify potential genes of interest to target, a comprehensive list of genes associated with mitochondrial disorders was compiled. The list was assembled in two ways. The first component included a high-evidence gene panel for mitochondrial disorders obtained from Genomics England PanelApp (Mitochondrial disorders panel v.4.0). We selected only the high-evidence panel (‘green list’), excluding the medium-evidence (‘amber list’) and low-evidence panels (‘red list’), to ensure the reliability of pathogenicity associations in the included genes. The second component involved a thorough review of the literature, where we included nuclear-encoded genes consistently reported to be involved in mitochondrial pathways. This way the target panel contained all known human disease genes encoding mitochondrial proteins including nuclear genes with involvement of mitochondrial function/dynamics or mutations in the mtDNA. The full list of genes is shown in Supplemental Materials.

### Whole exome sequencing (WES) reanalysis and reinterpretation

The cohort was created virtually, as previously described [35–37]. The data were reanalysed using a centralised, automated analysis, and filtering approach developed within the RD-Connect GPAP. Variants were filtered for those (a) located in genes associated with mitochondrial diseases (see list in Supplemental Materials), (b) Genome Aggregation Database (gnomAD) allele frequency of < 0.005, (c) internal RD-Connect GPAP allele frequency of < 0.02, (d) a HIGH or MODERATE putative effect prediction, as defined by SnpEFF, a genetic variant annotation and functional effect prediction toolbox, and (e) having zygosity compatible with the inheritance patterns of the gene (based on OMIM database) in which the variants were observed. Variants classified as benign or likely benign in ClinVar were excluded from further analysis. For single nucleotide variants (SNVs), a Combined Annotation Dependent Depletion (CADD) score above 20 was used as an additional criterion for pathogenicity assessment. It is important to note that base insertions, deletions, and duplications did not generate CADD scores and were evaluated using literature search and online tools such as ClinVar. In addition to consulting ClinVar and the ACMG guidelines, we also considered relevant literature surrounding each variant, when available, to further inform our assessment. In total, 1379 variants of interest were identified in 959 patients, of which 1326 variants were excluded for the following reasons: (a) previously reported as benign; (b) low pathogenicity prediction, as indicated by a CADD score of less than 20; (c) annotated in GPAP as solved with alternative genetic diagnosis; (d) incompatible inheritance pattern; (e) incompatible phenotype or non-neuropathic phenotype on further phenotype analysis; (f) incomplete HPO list resulting in ambiguous clinical diagnosis; and (g) conflicting reports of pathogenicity of variant in the literature. Additionally, we excluded variants of uncertain significance, where interpretation was not possible due to insufficient information. With this approach we aimed to maximise the chance that the included variants were likely to be causative.

## Results

### Cohort description

Of the 10,935 individuals on GPAP, a cohort of 2087 patients was created for those that had at least one HPO term suggestive of a possible neuropathic phenotype. Within this cohort, 1379 variants were identified in 959 patients. Of the 959 patients, 507 were male (52.87%), 415 were female (43.27%), and for 37 (3.86%) their gender was not reported.

Of the 1379 variants, 984 were heterozygous, 262 homozygous, 125 putative compound heterozygous, and 8 were located in the mitochondrial genome. Forty-four variants in 42 patients were found to be either previously reported as causative or identified here as likely to be causative of the patient’s disease. Approximately half of these cases (50%) had already been marked as solved in GPAP. Heterozygous variants were only considered to be potentially causative in genes with known autosomal dominant or X-linked dominant inheritance. Variants in genes with autosomal recessive inheritance were only considered causative if homozygous or compound heterozygous. Contact has been attempted with the involved centres to establish whether a causative variant has been identified, or to discuss the variant of interest.

### Variants identified in mitochondrial disease genes

**Fig. 2:**
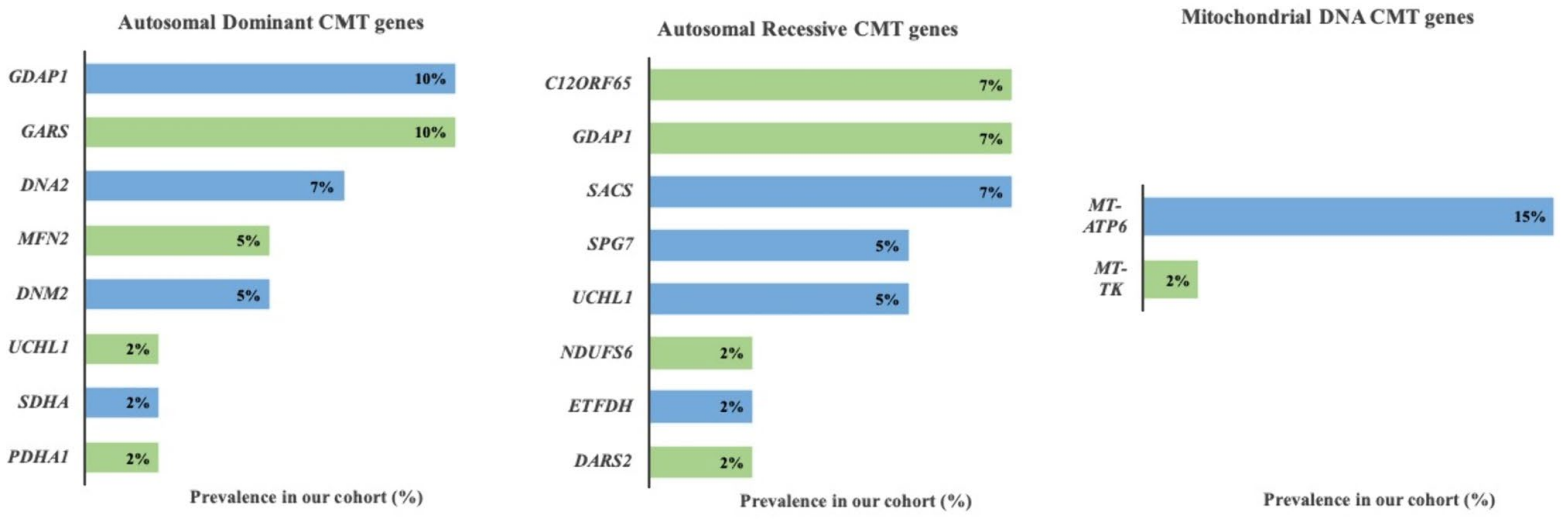
Prevalence of mitochondrial disease genes in the neuropathy cohort per inheritance pattern

The 44 included variants comprised 18 heterozygous, 14 homozygous, 4 putative compound heterozygous, and 8 mitochondrial variants (Tables 1, 2, 3). The genes responsible for each inheritance mode are shown in Fig. 2. These variants were present in 42 patients from 36 families, contributed by a total of 14 different GPAP submitter groups; a total of 322 HPO terms were registered, with an average of 6 terms per patient. The number of registered terms ranged from a minimum of 1 to a maximum of 16. Of the 42 patients included, 21 were male (50%), 20 were female (48%), and 1 patient’s gender was unknown (2%).

**Table 1:** - Pathogenic and likely pathogenic heterozygous nuclear variants causing autosomal dominant CMT in the virtual peripheral neuropathy cohort generated in GPAP

Patient	Sex	Gene	Variant	Protein
P1	Male	DNA2	c.2593dupT	p.Ser865PhefsTer21
P2	Female	DNA2	c.2593dupT	p.Ser865PhefsTer21
P3	Female	DNA2	c.2167G > A	p.Val723Ile
P4	Male	DNM2	c.1291dupG	p.Val431GlyfsTer52
P5	Male	DNM2	c.1291dupG	p.Val431GlyfsTer52
P6	Male	GARS	c.647A > G	p.His216Arg
P7	Male	GARS	c.1415A > G	p.His472Arg
P8	Female	GARS	c.1528A > C	p.Lys510Gln
P9	Female	GARS	c.1528A > C	p.Lys510Gln
P10	Female, aunt of P11	GDAP1	c.571C > T	p.Arg191Ter
P11	Male	GDAP1	c.571C > T	p.Arg191Ter
P12	Female, mum of P11	GDAP1	c.571C > T	p.Arg191Ter
P13	Female	GDAP1	c.617_618insG	p.Lys207GlufsTer4
P14	Female	MFN2	c.823C > T	p.Arg275Trp
P15	Male	MFN2	c.1126A > G	p.Met376Val
P16	Female	PDHA1	c.1019G > A	p.Arg340His
P17	Male	SDHA	c.91C > T	p.Arg31Ter
P18	Female	UCHL1	c.260_261dupGG	p.Asn88GlyfsTer28

**Table 2:** Pathogenic and likely pathogenic homozygous and presumed compound heterozygous nuclear variants causing autosomal recessive CMT in the virtual peripheral neuropathy cohort generated in GPAP

Patient	Sex	Gene	Variant	Protein
Homozygous				
P19	Male	C12ORF65	c.96_99dupATCC	p.Pro34IlefsTer25
P20	Female, sib of P19	C12ORF65	c.96_99dupATCC	p.Pro34IlefsTer25
P21	Male	C12ORF65	c.96_99dupATCC	p.Pro34IlefsTer25
P22	Male	DARS2	c.259G > A	p.Asp87Asn
P23	Male	ETFDH	c.1130 T > C	p.Leu377Pro
P24	Male	GDAP1	c.715C > T	p.Leu239Phe
P25	Female	GDAP1	c.786delG	p.Phe263LeufsTer22
P26	Male	GDAP1	c.712 T > G	p.Trp238Gly
P27	Male	NDUFS6	c.320_323delCAAA	p.Thr107LysfsTer40
P28	Male	SACS	c.7273C > T	p.Arg2425Ter
P29	Female	SACS	c.2182C > T	p.Arg728Ter
P30	Female	SACS	c.6634_6637delACAA	p.Thr2212SerfsTer7
P31	Male	UCHL1	c.629_631delGAG	p.Gly210del
P32	Female, sib of P31	UCHL1	c.629_631delGAG	p.Gly210del
Compound heterozygous				
P33	Female	SPG7	c.1045G > A	p.Gly349Ser
		SPG7	c.1529C > T	p.Ala510Val
P34	Female	SPG7	c.1454_1462delGGCGGGAGA	p.Arg485_Glu487del
		SPG7	c.1529C > T	p.Ala510Val

**Table 3:** Pathogenic and likely pathogenic mitochondrial DNA variants causing CMT in the virtual peripheral neuropathy cohort generated in GPAP

Patient	Gender	Gene	Variant	Protein
P35	Female, sib of P36	MT-ATP6	m.8993 T > C	p.Leu156Pro
P36	Female	MT-ATP6	m.8993 T > C	p.Leu156Pro
P37	Female	MT-ATP6	m.9176 T > C	p.Leu217Pro
P38	Male, sib of P40	MT-ATP6	m.9185 T > C	p.Leu220Pro
P39	Male	MT-ATP6	m.9185 T > C	p.Leu220Pro
P40	Male	MT-ATP6	m.9185 T > C	p.Leu220Pro
P41	Male	MT-ATP6	m.9185 T > C	p.Leu220Pro
P42	Male	MT-TK	m.8344A > G	N/A

We also identified 7 patients with HPO terms representing a peripheral neuropathy and heterozygous pathogenic or likely pathogenic *SPG7* mutations. The role of *SPG7* heterozygous variants in the aetiology of neuropathies is currently disputed. To gain a better insight whether this association is real, we tested the frequency of heterozygous *SPG7* variants in our neuropathy cohort, compared to the wider GPAP. We found that 1.097% (120 of 10,935) of GPAP participants harbour pathogenic or likely pathogenic heterozygous *SPG7* variants, while only 0.240% (5 out of 2,087) of our neuropathy cohort. Consequently, this finding does not support the role of heterozygous *SPG7* mutations in the aetiology of mitochondrial neuropathies. It is worth noting that RD-Connect is made up of mostly affected individuals and not healthy controls; therefore, it is not accurately demonstrating the prevalence of reportedly pathogenic heterozygous *SPG7* mutations in a healthy population.

## Discussion

In this study, our aim was to investigate the occurrence of mitochondrial disease genes in patients with CMT. “Mitochondrial CMT” has been previously reported to be caused by a number of mitochondrial disease genes [5], suggesting a link between mitochondrial dysfunction and neuropathies. While certain forms of mitochondrial CMT, such as MFN2, SANDO, and MNGIE, are well documented, and have been detected by candidate gene sequencing in the past, the mechanism leading to a predominant neuropathy in some individuals remains unclear.

To explore the potential presence of additional mitochondrial forms of CMT, we conducted a search using RD-Connect GPAP. We created a virtual cohort of patients classified by HPO terms typical for CMT and we searched for causative variants in all known mitochondrial disease genes. Our search identified 1,379 variants in 93 genes affecting a total of 959 patients. Further analysis showed that only 44 of these variants, in 16 disease genes, were potentially causative affecting 42 patients in 36 families. In addition to some known variants in genes already known to be associated with neuropathy such as *GDAP1, GARS1, DNM2, MFN2, SACS, C12orf65, SPG7, SDHA, PDHA1*, and *MT-ATP6,* we detected some variants in unexpected genes such as *DNA2, NDUFS6, ETFDH, SDHA, DARS2* and *UCHL1,* and *MT-TK* highlighting that neuropathy presents more widely in mitochondrial disease.

Mutations of *SPG7* are predominantly autosomal recessive; however, heterozygous *SPG7* mutations have also been reported in association with diverse clinical manifestations such as optic atrophy, spastic paraplegia, and peripheral neuropathy [38]. The putative role of these heterozygous *SPG7* variants in the aetiology of neuropathies remains controversial. In an attempt to elucidate this association further, we assessed the frequency of heterozygous *SPG7* variants within our neuropathy cohort. We observed a reduced frequency of heterozygous *SPG7* mutation carriers within our cohort compared to the rest of the samples we could access in GPAP. This decreased prevalence in our neuropathy cohort does not support the pathogenic role of heterozygous *SPG7* mutations in the aetiology of mitochondrial neuropathies. However, we detected biallelic *SPG7* variants in 3 patients suggest the association of *SPG7* with autosomal recessive peripheral neuropathy.

By exploring the genetic landscape of peripheral neuropathies and identifying potential causative variants in genes with mitochondrial mechanisms, we contribute to a deeper understanding of the molecular mechanisms underlying this complex disease. Our findings underscore the utility of large-scale data analysis and the value of reanalysing exome/genome data over time as new genetic associations are reported and NGS technologies evolve. We also emphasise the importance of comprehensive phenotypic characterisation of patients by clinicians to assist future cohort studies and increase the yield of a genetic diagnosis.

Whole exome sequencing (WES) has emerged as a valuable diagnostic tool [18], but even with its application, a substantial number of patients remain undiagnosed. The diagnostic yield from WES varies depending on the setting and inclusion criteria, ranging from 15 to 60% [39]. To address this diagnostic gap, before performing new genomic testing (whole genome sequencing, RNA sequencing, proteomics, etc.), expert reanalysis of WES data holds promise for identifying missed or newly discovered genetic variants [18, 40].

This study has important limitations that should be acknowledged. Firstly, there is a possibility of missed patients due to incomplete or incorrect HPO term entry by the uploading clinician. Although efforts were made to ensure accurate phenotypic characterisation, variations in data entry may have resulted in some patients being inadvertently excluded from the analysis. Secondly, gene segregation could not be performed in cases where only index patient data were available, as opposed to a trio. Attempts were made to contact all centres whose variants were included in this study to ascertain if the patient had been previously diagnosed or to discuss the variant identified. Moreover, the platform does not currently enable the efficient cohort-search feature for mitochondrial encoded genes, meaning it was necessary to select individual variants within the mitochondrial genome, which could lead to potential omissions.

Additionally, despite the potential benefits of reanalysis, several challenges and limitations need to be considered. The interpretation of variants can be complex and may vary between different laboratories or databases, leading to discrepancies in variant classification. Updates in variant databases and evolving guidelines for variant interpretation also demand regular re-evaluation of previously reported variants. Furthermore, the identification of disease-causing genes or variants may require functional studies or additional evidence beyond bioinformatic analysis. Lastly, the dynamic nature of genetic research requires continuous data monitoring and re-evaluation as new genetic associations, and disease mechanisms emerge. Despite these challenges, reanalysis provides an opportunity to enhance diagnostic yield and contribute to the evolving understanding of rare diseases, ultimately improving patient care and management.

In conclusion, inherited neuropathies are rare and complex disorders affecting the peripheral nerves, and mitochondria play a crucial role in the disease. This study shows how frequently is CMT caused by mutations in primary mitochondrial disease genes. This study demonstrates the potential of RD-Connect as a powerful tool for unravelling the underlying causes of rare diseases, including inherited neuropathies, and highlights the importance of international collaboration to improve the diagnosis and treatment of rare diseases.

### Supplementary Information

Below is the link to the electronic supplementary material.

Supplementary file1 (DOCX 19 KB)

## Data Availability

All genomic data are deposited in the RD-Connect Genome Phenome Analysis Database (GPAP).
