# Supplementary material for: Variants in mitochondrial disease genes are common causes of inherited peripheral neuropathies
Source: J Neurol. 2024 Mar 28;271(6):3546–53. doi: 10.1007/s00415-024-12319-y (PMC11136726; doi:10.1007/s00415-024-12319-y)
Supplement: Supplementary file 1 — Supplementary file1 (DOCX 19 KB) [file 415_2024_12319_MOESM1_ESM.docx]

**Supplemental material**

a) Neuropathy Cohort HPO list

1. Acute episodes of neuropathic symptoms HP:0003489
2. Demyelinating peripheral neuropathy HP:0007108
3. Distal muscle weakness HP:0002460
4. EMG: neuropathic changes HP:0003445
5. Entrapment neuropathy HP:0012181
6. Fatigable weakness HP:0003473
7. Limb muscle weakness HP:0003690
8. Motor polyneuropathy HP:0007178
9. Muscle weakness HP:0001324
10. Peripheral axonal neuropathy HP:0003477
11. Peripheral neuropathy HP:0009830
12. Proximal muscle weakness HP:0003701
13. Sensory ataxic neuropathy HP:0003434
14. Sensory axonal neuropathy HP:0003390
15. Sensory neuropathy HP:0000763
16. Weakness due to upper motor neuron dysfunction HP:0010549
17. EMG abnormality HP:0003457
18. EMG: axonal abnormality HP:0003482
19. EMG: positive sharp waves HP:0030007
20. EMG: repetitive nerve stimulation abnormality HP:0030000
21. EMG: slow motor conduction HP:0100287
22. Mixed demyelinating and axonal polyneuropathy HP:0007327
23. Motor axonal neuropathy HP:0007002
24. Peripheral axonal atrophy HP:0003384
25. Peripheral axonal degeneration HP:0000764
26. Single fibre EMG abnormality HP:0030006
27. Distal sensory impairment HP:0002936
28. Distal upper limb muscle weakness HP:0008959
29. Foot dorsiflexor weakness HP:0009027
30. Areflexia of lower limbs HP:0002522
31. Abnormal activity of mitochondrial respiratory chain HP:0011922
32. Hand muscle atrophy HP:0009130
33. Demyelinating motor neuropathy HP:0007220

b) Neuropathy cohort target genes

| AARS2 | COX14 | HARS2 | MRPS34 | PET100 | SURF1 |
| --- | --- | --- | --- | --- | --- |
| ABAT | COX20 | HCCS | MSTO1 | PMPCA | TAZ |
| ABCB7 | COX6A1 | HIBCH | MTFMT | PMPCB | TIMM50 |
| ACAD9 | COX7B | HLCS | MTO1 | PNPLA8 | TIMM8A |
| ACO2 | CYC1 | HSD17B10 | MTPAP | PNPT1 | TMEM126B |
| AFG3L2 | DARS2 | HSPD1 | NADK2 | POLG | TOP3A |
| AGK | DLAT | HTRA2 | NARS2 | PPA2 | TPK1 |
| AIFM1 | DLD | IARS2 | NAXE | PUS1 | TRIT1 |
| ANO10 | DNA2 | IBA57 | NDUFA1 | QRSL1 | TRMT10C |
| APOPT1 | DNAJC19 | ISCA1 | NDUFA4 | RARS2 | TRMT5 |
| APTX | DNM1L | ISCA2 | NDUFA6 | RMND1 | TRMU |
| ATAD3A | DNM2 | ISCU | NDUFA9 | RNASEH1 | TRNT1 |
| ATP5D | EARS2 | KARS | NDUFAF2 | RTN4IP1 | TSFM |
| BOLA3 | ECHS1 | LARS2 | NDUFAF5 | SACS | TUFM |
| BTD | ELAC2 | LIAS | NDUFAF6 | SARS2 | UCHL1 |
| C12orf65 | ETFDH | LIPT1 | NDUFAF8 | SDHA | UQCC2 |
| C19orf70 | ETHE1 | LIPT2 | NDUFB11 | SDHAF1 | UQCRB |
| C1QBP | FARS2 | LONP1 | NDUFB3 | SDHD | VARS2 |
| CA5A | FBXL4 | LRPPRC | NDUFB8 | SERAC1 | WARS2 |
| CARS2 | FDX2 | LYRM7 | NDUFS6 | SFXN4 | YARS2 |
| CHCHD10 | FDXR | MARS2 | NDUFV2 | SLC19A2 |  |
| CLP1 | FH | MDH2 | NFU1 | SLC19A3 |  |
| CLPB | FIG4 | MECR | OPA1 | SLC25A1 |  |
| CLPP | FLAD1 | MFF | OPA3 | SLC25A12 |  |
| COA6 | FOLR1 | MFN2 | PARS2 | SLC25A19 |  |
| COA7 | FOXRED1 | MGME1 | PC | SLC25A26 |  |
| COQ2 | GARS | MICU1 | PDHA1 | SLC25A3 |  |
| COQ4 | GDAP1 | MIPEP | PDHB | SLC25A32 |  |
| COQ6 | GFER | MPC1 | PDHX | SLC25A38 |  |
| COQ7 | GFM1 | MRPL3 | PDK3 | SLC25A42 |  |
| COQ8A | GFM2 | MRPL44 | PDP1 | SLC25A46 |  |
| COQ8B | GLRX5 | MRPS2 | PDSS1 | SORD |  |
| COQ9 | GTPBP3 | MRPS22 | PDSS2 | SPG7 |  |
